# MicroRNA‐122 and cytokeratin‐18 have potential as a biomarkers of drug‐induced liver injury in European and African patients on treatment for mycobacterial infection

**DOI:** 10.1111/bcp.14736

**Published:** 2021-01-26

**Authors:** Sarah A.E. Rupprechter, Derek J. Sloan, Wilna Oosthuyzen, Till T. Bachmann, Adam T. Hill, Kevin Dhaliwal, Kate Templeton, Joshua Matovu, Christine Sekaggya‐Wiltshire, James W. Dear

**Affiliations:** ^1^ Pharmacology, Therapeutics and Toxicology, Centre for Cardiovascular Science University of Edinburgh, The Queen's Medical Research Institute Edinburgh UK; ^2^ School of Medicine University of St Andrews St Andrews UK; ^3^ Chancellor's Building Edinburgh University/Infection Medicine Edinburgh UK; ^4^ The Queen's Medical Research Institute Edinburgh University/Centre for Inflammation Research Edinburgh UK; ^5^ Infectious Diseases Institute Makerere University College of Health Sciences Kampala Uganda

**Keywords:** cytokeratin‐18, drug‐induced liver injury, microRNA‐122tuberculosis

## Abstract

**Aims:**

Patients on antituberculosis (anti‐TB) therapy are at risk of drug‐induced liver injury (DILI). MicroRNA‐122 (miR‐122) and cytokeratin‐18 (K18) are DILI biomarkers. To explore their utility in this global context, circulating miR‐122 and K18 were measured in UK and Ugandan populations on anti‐TB therapy for mycobacterial infection.

**Methods:**

Healthy subjects and patients receiving anti‐TB therapy were recruited at the Royal Infirmary of Edinburgh, UK (ALISTER—ClinicalTrials.gov Identifier: NCT03211208). African patients with human immunodeficiency virus–TB coinfection were recruited at the Infectious Diseases Institute, Kampala, Uganda (SAEFRIF—NCT03982277). Serial blood samples, demographic and clinical data were collected. In ALISTER samples, MiR‐122 was quantified using polymerase chain reaction. In ALISTER and SAEFRIF samples, K18 was quantified by enzyme‐linked immunosorbent assay.

**Results:**

The study had 235 participants (healthy volunteers [*n =* 28]; ALISTER: active TB [*n =* 30], latent TB [*n =* 88], nontuberculous mycobacterial infection [*n =* 25]; SAEFRIF: human immunodeficiency virus‐TB coinfection [*n =* 64]). In the absence of DILI, there was no difference in miR‐122 and K18 across the groups. Both miR‐122 and K18 correlated with alanine transaminase (ALT) activity (miR‐122: *R* = .52, 95%CI = 0.42–0.61, *P <* .0001. K18: *R* =0.42, 95%CI = 0.34–0.49, *P <* .0001). miR‐122 distinguished those patients with ALT>50 U/L with higher sensitivity/specificity than K18. There were 2 DILI cases: baseline ALT, 18 and 28 IU/L, peak ALT 431 and 194 IU/L; baseline K18, 58 and 219 U/L, peak K18 1247 and 3490 U/L; baseline miR‐122 4 and 17 fM, peak miR‐122 60 and 336 fM, respectively.

**Conclusion:**

In patients treated with anti‐TB therapy, miR‐122 and K18 correlated with ALT and increased with DILI. Further work should determine their diagnostic and prognostic utility in this global context‐of‐use.

**What is already known about this subject**

- Drug‐induced liver injury (DILI) is a major concern in the treatment of tuberculosis (TB).
- There are new biomarkers for DILI (microRNA‐122 and cytokeratin‐18) that have the potential to add value because of enhanced specificity/sensitivity compared to current tests.
- These mechanistically informative DILI biomarkers have qualifying data from large USA and European studies. However, these markers have not been tested in Africa. Given the global burden of TB our aim was to explore biomarker utility in this global context of use.

**What this study adds**

- The baseline concentrations of cytokeratin‐18 did not substantially differ across UK and Ugandan populations on treatment for TB and human immunodeficiency virus. MicroRNA‐122 did not differ across healthy subjects and UK patients treated for mycobacterial infection.
- These biomarkers significantly correlated with alanine transaminase activity (current liver injury marker) and were elevated with DILI. MicroRNA‐122 distinguished those patients with alanine transaminase >50 U/L with higher sensitivity/specificity than K18.
- MicroRNA‐122 and cytokeratin‐18 have potential in this context‐of‐use and should be taken forward into larger studies, which could provide data for formal qualification.

## 1 INTRODUCTION

Tuberculosis (TB) is in the top 10 global causes of death, with an estimated 10 million new cases, 1.2 million deaths among human immunodeficiency virus (HIV)‐negative individuals and 251 000 deaths among HIV‐positive individuals in 2018.^1^ The global burden of TB is unequally distributed, disproportionately affecting low‐ and middle‐income countries particularly in Africa and Asia. One of the barriers to the effective treatment of TB are the adverse drug reactions experienced by patients on anti‐TB medications, with 1 of the most common being drug‐induced liver injury (DILI).^2^ Three of the 4 first‐line drugs used in the treatment of TB—isoniazid, rifampicin and pyrazinamide—are potentially hepatotoxic.^3^ The estimate of the incidence of DILI in individuals undergoing anti‐TB treatment for active TB varies from 2 to 33% depending on the cohort studied, drug regimen used, monitoring and reporting practices.^2^, ^4^, ^5^ Individuals who experience DILI often need to stop treatment and, if clinically indicated, recommence once liver function tests (LFTs) return to normal. However, for some individuals, re‐exposure to the same drugs leads to reoccurrence of DILI,^6^ and for others, liver injury progresses even after treatment has stopped.^7^ Therefore, there is an unmet clinical need for new tools to improve the safety of this essential antimicrobial treatment.

Diagnosis of DILI relies on LFTs, with alanine aminotransferase (ALT) activity considered a gold standard for determining liver injury. The DILI Expert Working Group defines DILI as ≥3× upper limit of normal (ULN) of ALT in the presence of symptoms, or ≥5× ULN ALT in the absence of symptoms.^8^ Although ALT is currently the gold standard for determining DILI, there are issues associated with its use. ALT is not specific to the liver and can provide false positive results, with elevations in ALT occurring after muscular damage following exercise^9^ or subsequent to a myocardial infarction.^10^ Furthermore, elevations in ALT are not specific to DILI^11^ and can occur due to metabolic perturbations.^12^, ^13^ In paracetamol‐overdose DILI, there is a delay between insult to the liver and rise in ALT,^14^ meaning ALT is not optimal as a biomarker of DILI in this context. To address these challenges recent work has identified novel biomarkers capable of diagnosing, and in some cases predicting DILI.

MicroRNAs (miRNAs) are small noncoding RNAs regulate post‐transcriptional gene expression. MiR‐122 is a 22‐nucleotide microRNA is highly expressed in, and highly specific for, the liver, with little to no expression in other tissues. In liver injury, miR‐122 is released from necrotic hepatocytes, resulting in elevated miR‐122 concentrations in the bloodstream.^15^ Cytokeratin‐18 (K18) is an intermediate filament protein responsible for maintaining the cytoskeletal structure in the liver and other epithelial cells and is reported to make up 5% of the liver's total protein content.^16^ K18 is a mechanistic biomarker of liver injury, providing information on the pattern of cell death. In apoptosis, the release of a caspase‐cleaved form of K18 (cc‐K18) is an early event during cellular structural rearrangement,^17^ whereas, in necrosis, the full‐length form of K18 (FL‐K18) is passively released upon cell death.^17^ MiR‐122 and K18 are able to predict DILI in patients who overdose on paracetamol earlier than standard LFTs^18^, ^19^ and provides enhanced hepatic specificity over other biomarkers.^20^ Both novel biomarkers have regulatory support from the US Food and Drug Administration as biomarkers for DILI,^21^ although to date their development has been largely limited to testing in western populations.

The aim of this study was to explore the properties of miR‐122 and K18 in relevant European and African patients with mycobacterial infection (with and without HIV coinfection). Specifically, our aims were to determine whether the infective disease process and routine management with anti‐TB medicines affect these biomarkers in the absence of DILI and to characterise how miR‐122 and K18 change in relation to ALT and in cases of DILI.

## 2 METHODS

Participants with active TB, latent TB and nontuberculous mycobacterial (NTM) infection were recruited into the Assessing Antibiotic‐Induced Liver Injury for the Stratification of Tuberculosis Patients (ALISTER) clinical study at the Royal Infirmary, Edinburgh (ClinicalTrials.gov Identifier: NCT03211208). Participants with HIV‐TB coinfection were recruited into the Safety and Efficacy of High Dose Rifampicin in Tuberculosis (TB)‐HIV Co‐infected Patients on Efavirenz‐ or Dolutegravir‐based Antiretroviral Therapy (SAEFRIF) clinical trial at the Infectious Disease Institute, Kampala, Uganda (ClinicalTrials.gov Identifier: NCT03982277).

### 2.1 Healthy subjects

As a control group to test whether circulating miR‐122 and K18 were affected by active TB, latent TB or NTM infection, adults with no medical complaints and no medication use were recruited and blood was drawn with informed consent.

### 2.2 ALISTER clinical study

Participants were recruited at the Royal Infirmary of Edinburgh. Adults (≥16 years, ≤85 years), receiving treatment for active or latent TB, or NTM infection were included. Patients were excluded if they did not have the capacity to provide informed consent or were known to be HIV positive. Full written informed consent was obtained from every participant and the study was approved by the West of Scotland Research Ethics Committee.

Patients were classified as having active TB either if they had culture confirmation of *Mycobacterium tuberculosis* and presence of active disease, or if a clinician decided there was sufficient evidence of active disease to start them on treatment. Latent TB patients had a positive interferon‐γ release assay and no evidence of active disease. Patients with NTM infection had grown at least 2 cultures with nontuberculous mycobacterium and had clinical signs of pulmonary disease.^22^, ^23^

Active and latent TB patients were treated following World Health Organisation guidelines.^24^, ^25^ Patients with susceptible active TB were treated with isoniazid, rifampicin, ethambutol and pyrazinamide for an initiation phase of 2 months, followed by rifampicin and isoniazid for a continuation phase of 4 months.^24^ Patients with latent TB were treated with either a combination of isoniazid and rifampicin for 3 months, or isoniazid or rifampicin alone for 6 months.^25^ Patients with *Mycobacterium avium* complex infection were treated with the recommended regimen of rifampicin, ethambutol and clarithromycin for 2 years.^23^ Dependent on the NTM species, disease severity, resistance profile of the infection and tolerance of the individual, drugs were replaced or added, including isoniazid, moxifloxacin, azithromycin and amikacin.^22^, ^23^

### 2.3 SAEFRIF clinical trial

The SAEFRIF clinical trial (NCT03982277) was performed at the Infectious Disease Institute at Makerere University, Uganda. The data presented in this paper provide preliminary data on liver safety from 64 trial participants. A full report on predefined study end‐points will be provided on trial completion. Ethical approval for this study was sought from Joint Clinical Research Council ethics committee, the National Drug Authority and the Uganda National Council for Science and Technology. All participants signed an informed consent form prior to study enrolment.

Inclusion criteria were HIV‐infected patients aged ≥18 years who were due to initiate rifampicin‐containing therapy for newly diagnosed active TB, and were either already taking or planning to start efavirenz‐based or dolutegravir‐based antiretroviral therapy. Exclusion criteria were patients who have rifampicin resistant TB, pregnant women, women of reproductive age on dolutegravir who decline the use of effective contraceptive, patients with liver disease, ALT > 5× ULN or glomerular filtration rate < 50 mL/min.

The trial protocol is described by Nabisere et al.^26^ Patients were randomised to 1 of 4 arms of the trial for the first 2 months of treatment, either the standard (10 mg/kg) or high dose (35 mg/kg) rifampicin alongside standard doses of the other first line TB drugs and either efavirenz‐ or dolutegravir‐based antiretroviral therapy. Baseline blood samples (serum) were collected at baseline and weeks 2, 4, 6 and 8 of treatment.

### 2.4 Data and blood samples

For the ALISTER study and SAEFRIF trial demographic and clinical data from the participants were recorded from medical records and clinical trial records. Liver function test results (ALT) were recorded from each clinic visit. Blood samples were collected at first clinic visit and subsequent clinic visits. Once collected, blood was processed by centrifugation and the supernatant was aliquoted and stored at −80°C.

### 2.5 Quantification of miR‐122

Serum samples were stored at −80°C before analysis. Freeze–thaw cycles were avoided to preserve miRNA integrity. MiR‐122 was quantified in samples from healthy volunteers and the ALISTER study. miR‐122 could only be measured in Edinburgh. Due to the global COVID pandemic and the HIV‐positive status of the SAEFRIF samples these could not be shipped and miR‐122 was not measured. All ALISTER samples were stored for a maximum of 1 year (median [interquartile range, IQR]: 16.3 [7.6–33.3] weeks). This is less time in storage than previously published studies, which have demonstrated miRNA stability.^27^, ^28^ MiRNA was extracted using the miRNeasy Serum/Plasma kit (Qiagen, Venlo, Netherlands) following the manufacturer's instructions. Total RNA was extracted from 50 μL diluted in 150 μL nuclease free water. Briefly, RNA was extracted from the serum by lysis reagent (1000 μL) and chloroform (200 μL). After centrifugation at 12 000 × *g* for 15 minutes at 4°C up to 600 μL of the aqueous phase was transferred to a new tube with 900 μL absolute ethanol. RNA was purified on a RNeasy minElute spin column and eluted in 15 μL RNase‐free water and stored at −80°C. Extraction efficiency was monitored by adding 5.6 × 10^8^ copies of synthetic *Caenorhabditis elegans* miR‐39 spike‐in control after the addition of lysis reagent before the addition of chloroform and phase separation. The miScript II Reverse Transcription kit was used to prepare cDNA according to the manufacturer's instructions. Briefly, 2.5 μL of RNA eluate was reverse transcribed into cDNA. The synthesised cDNA was diluted and used for cDNA template in combination with the miScript SYBR Green polymerase chain reaction (PCR) kit (Qiagen, Venlo, The Netherlands) using the specific miScript assays (Qiagen, Venlo, The Netherlands). Reverse transcription (RT)‐PCR was performed in duplicate on a Light Cycler 480 (Roche, Burgess Hill, UK) using the recommended miScript cycling parameters. In this study, miR‐122 was quantified in fM by generating a standard curve. Serial dilutions of known standard were made using synthetic miR‐122 (syn hsa‐miR‐122‐5p, 219600, Qiagen). The dilutions were prepared in triplicate, using the same RT and PCR protocol as described above. The cycle threshold (Ct) values were plotted against the logarithm of the concentration, demonstrating a clear linear relationship between Ct value and Log (conc.). The Ct is defined as the number of PCR cycles required for the fluorescent signal to cross the threshold (exceed background level). The resultant regression line was used to ascertain the concentration of miR‐122 present in the samples. Acceptable repeatability was demonstrated by measuring the intra‐assay variability of miR‐122 duplicates^29^ and expressed as concentration (fM) per MIQE guidelines^30^ (CV: 0.25%, [0.11–0.49% IQR]). The intra‐assay variation in miR‐39 Ct values was assessed (CV median [IQR]: 3.04 [2.50–3.64] %). Reproducibility was determined by measuring interassay variability across plates and days by measuring miR‐122 concentrations of reference samples. A no‐enzyme control, omitting the reverse transcriptase enzyme during reverse transcription, and no‐template control omitting the cDNA in the RT‐PCR plate were also included in every run. No‐enzyme and no‐template controls had Ct values of >35. Ct values <35 were regarded as positive amplification signals.

### 2.6 Quantification of K18

Samples were stored at −80°C before analysis. K18 was quantified in samples from healthy volunteers and patients in the ALISTER study, with these samples stored for a maximum of just over 2 years (113 wk; median [IQR]: 48.4 [28.7–77.8] wk) prior to analysis. The K18 assay was established and samples were analysed by author S.A.E.R. in Uganda. K18 was quantified in serum samples from the SAEFRIF trial, these samples were stored for a maximum of 1 year (median [IQR]: 19.1 [11.1–26.2] wk). K18 has been reported as stable in serum up to 7 freeze–thaw cycles^31^ and for up to 2 years when frozen.^32^ Total K18 was quantified using the Peviva M65 classic enzyme‐linked immunosorbent assay (Bioaxxes, Tewkesbury, Glos, UK), according to the manufacturer's instructions. Samples were measured in duplicate. CV values for the dataset were (median [IQR]: 2.85% [1.33–4.97%]). Reproducibility was determined using the provided plate controls.

### 2.7 Assessment of causality

DILI was predefined in this study as >3× ULN ALT in the presence of symptoms or >5× ULN in absence of symptoms.^8^ The ULN for ALT was 50 IU/L in the populations studied, as defined by local clinical practice. The Roussel Uclaf Causality Assessment Method (RUCAM) was used to determine formal causality between anti‐TB medication and liver injury.^33^ The pattern of liver injury was determined using the R ratio, considering ALT and alkaline phosphatase activity. Further factors considered include time to onset, course of injury, risk factors (age and alcohol), concomitant drugs, the exclusion of nondrug causes of injury and previous information on drug hepatotoxicity.

### 2.8 Statistical analysis

Data were summarised as median (IQR) or *n* (%) for summary statistics of the study participants. One‐way Kruskal–Wallis ANOVA was used to determine the difference in miR‐122 and K18 between the healthy subjects and different patient groups. Wilcoxon matched‐pairs signed rank test was used to determine the difference in miR‐122 and K18 upon starting treatment. The coefficient of variation was calculated across 3 or more samples to assess the intraindividual variability of ALT and K18 in the HIV‐TB coinfected population. Correlation of the biomarkers was determined using Spearman's rank correlation. The difference between miR‐122 or K18 in samples grouped by ALT was determined using a Mann–Whitney t‐test. A receiver operating characteristic (ROC) analysis was undertaken on all patient samples grouped by normal ALT (≤50 IU/L) and elevated ALT (>50 IU/L). Statistical analyses were performed using Graphpad Prism (GraphPad Software, La Jolla, California).

## 3 RESULTS

**FIGURE 1:**
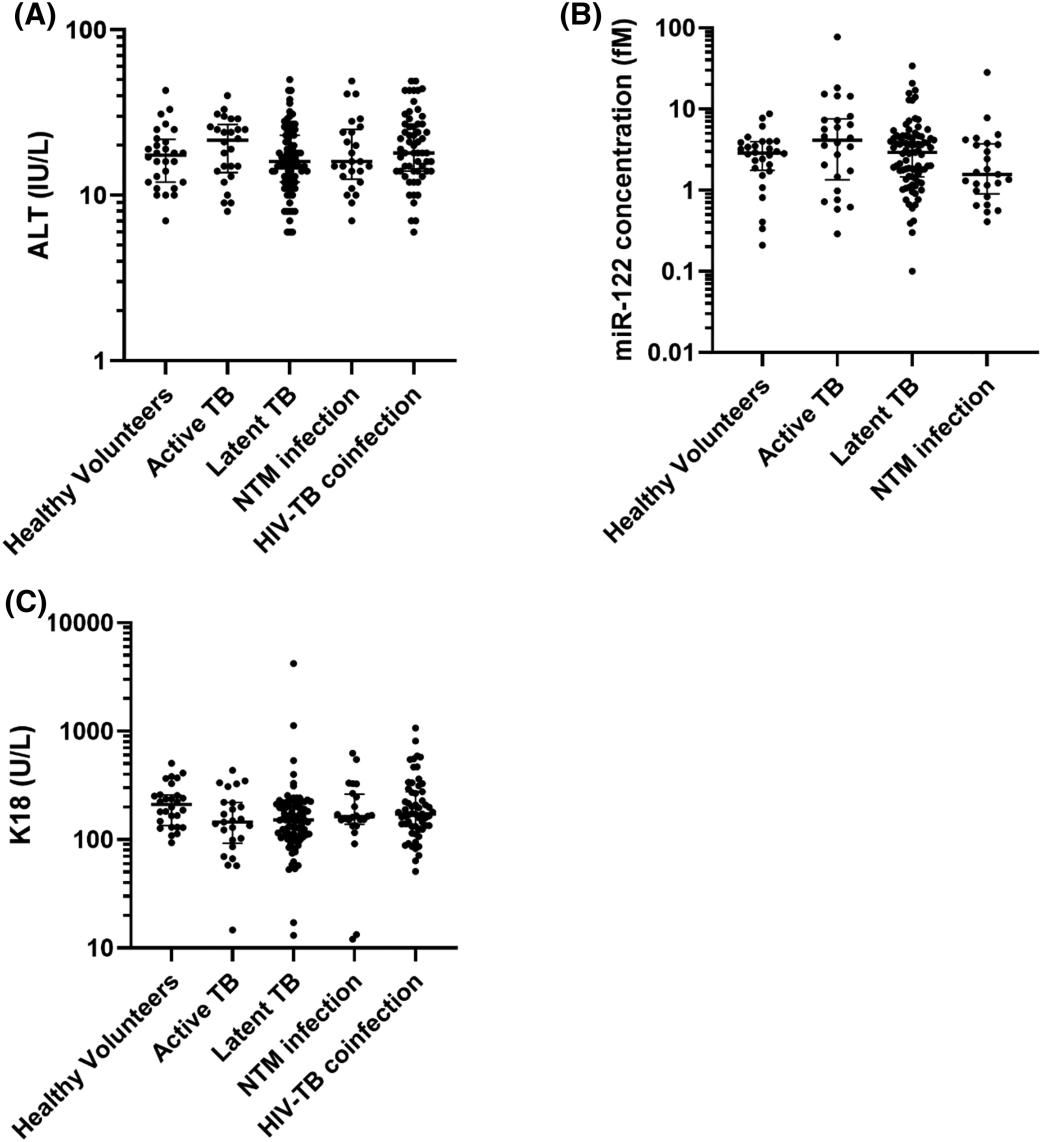
Circulating concentration of A, alanine transaminase (ALT; IU/l), B, microRNA (miR)‐122 (fM) and C, K18 (U/L). Data are the first collected samples from the ALISTER study or SAEFRIF trial. Participants include healthy volunteers (*n =* 28), active tuberculosis (TB; *n =* 26), latent TB (*n =* 87), nontuberculous mycobacteria (NTM) infection (*n =* 25) and human immunodeficiency virus (HIV)–TB coinfection (*n =* 59). Data are presented as dot plots. Line shows median and bars show interquartile range. The significance of differences between groups were determined by 1‐way Kruskal–Wallis ANOVA (ALT *P =* .2; miR‐122 *P =* .09; K18 *P =* .03)

**FIGURE 2:**
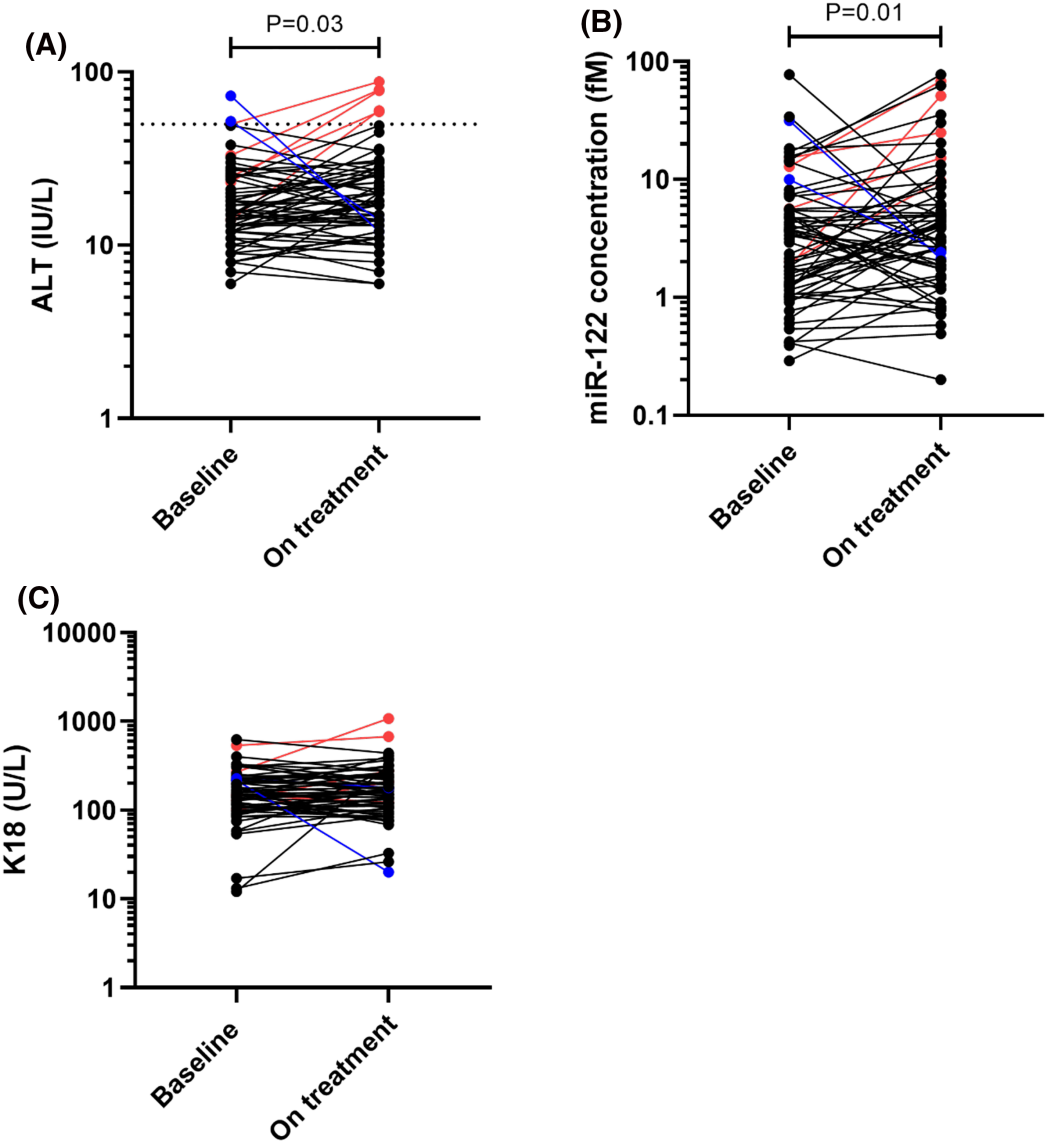
Circulating concentration of A, alanine transaminase (ALT; IU/L), B, microRNA (miR)‐122 (fM) and C, K18 (U/L) in sequential samples in patients within the ALISTER study, (active tuberculosis, *n =* 9; latent tuberculosis, *n =* 46; nontuberculous mycobacteria, *n =* 10) Data shown as dot plots. Black dots show patients with normal ALT activity at baseline and on treatment; blue dots show patients with ALT activity >50 U/L at baseline which decreased on treatment; red dots show patients whose ALT increased above 50 U/L with treatment. Dotted line on (A) ALT = 50 IU/L. The significance of differences between baseline and on treatment concentrations of biomarkers was determined by Wilcoxon signed rank test (ALT *P =* .03; miR‐122 *P =* .01; K18 *P =* .4)

**FIGURE 3:**
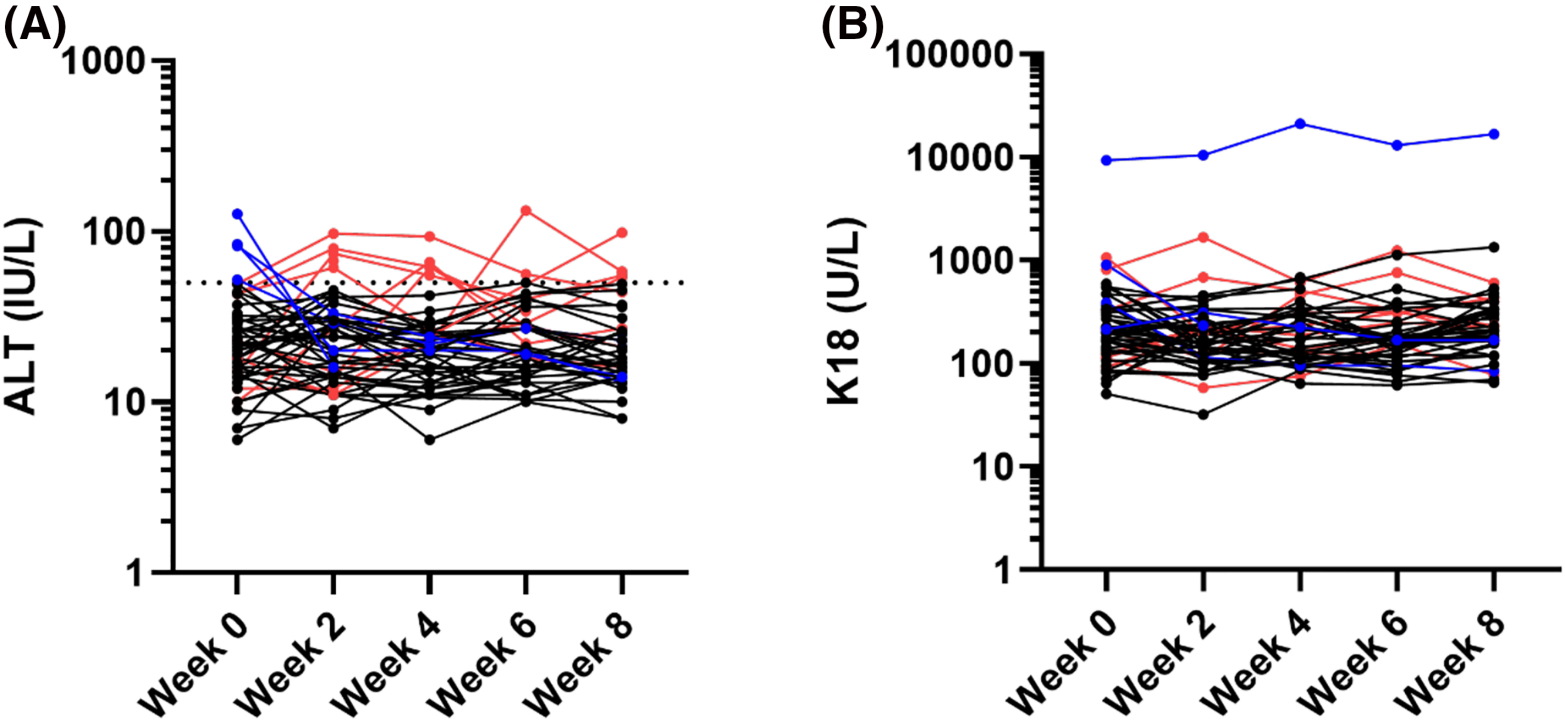
Circulating concentration of A, alanine transaminase (ALT; IU/L) and B, K18 (U/L) for sequential samples in patients within the SAEFRIF trial. Black dots show patients with normal ALT throughout treatment, red points show patients whose ALT rises >50 IU/L during treatment, blue points show patients whose ALT falls from >50 IU/L upon starting treatment. Dotted line on (A) ALT = 50 IU/L

A total of 207 patients were recruited into the study, along with 28 healthy volunteers; in ALISTER: active TB (*n =* 30), latent TB (*n =* 88), NTM infection (*n =* 25); in SAEFRIF: HIV‐TB coinfection (*n =* 64). The healthy volunteers had a median age of 27 years (IQR 24–30) and 64% were male. All were white British. Demographic and clinical characteristics for patients within ALISTER and SAEFRIF are presented in Table 1. There was no significant difference in miR‐122 (*P =* .09) between the groups. There was a statistically significant but clinically minor difference in K18 between groups (*P =* .03; Table 2, Figure 1). Serial samples were collected in 65 patients in the ALISTER study (Figure 2). The time patients had spent on treatment was median (IQR): active TB 3.0 (2.4–6.9); latent TB 3.9 (2.4–12.2); NTM infection 4.0 (2.2–6.9) weeks. Commencing treatment was associated with a statistically significant, clinically insignificant increase in ALT (median [IQR]: baseline 15 [12–24]; on treatment 17 [13–27] IU/L; *P =* .03) and miR‐122 (median [IQR]: baseline 3.12 [1.20–5.63]; on treatment 3.95 [1.75–7.98] fM; *P =* .01). There was also an increase in K18, which did not reach statistical significance (median [IQR]: baseline 150 [103–224]; on treatment 167 [110–246] U/L; *P =* .4). Serial samples were also collected in 50 SAEFRIF patients (Figure 3). In the SAEFRIF patients, starting treatment did not lead to a significant change in ALT (median [IQR]: week 0: 22 [14–31]; week 2: 24 [14–32] IU/L; *P =* .62) or K18 (median [IQR]: week 0: 204 [130–344]; week 2: 169 [117–262] U/L; *P =* .3).

**TABLE 1:** Clinical and demographic characteristics of patients in the study. Data are presented as median (interquartile range) or *n* (%)

		Active TB (*n =* 30)	Latent TB (*n =* 88)	NTM infection (*n =* 25)	HIV‐TB coinfection (*n =* 64)
Age (y)		41 (33–56)	40 (23–63)	66 (57–74)	38 (32–44)
Sex	**Male**	20 (67%)	23 (26%)	10 (40%)	40 (62%)
	**Female**	10 (33%)	65 (74%)	15 (60%)	24 (38%)
Ethnicity	**African**	2 (7%)	7 (8%)	0 (0%)	64 (100%)
	**Arab**	0 (0%)	1 (1%)	0 (0%)	0 (0%)
	**Bangladeshi**	1 (3%)	2 (2%)	0 (0%)	0 (0%)
	**Indian**	7 (23%)	3 (3%)	0 (0%)	0 (0%)
	**Iraqi**	1 (3%)	0 (0%)	0 (0%)	0 (0%)
	**Pakistani**	5 (17%)	3 (3%)	1 (4%)	0 (0%)
	**South‐‐east Asian**	2 (7%)	1 (1%)	0 (0%)	0 (0%)
	**White**	12 (40%)	66 (75%)	24 (96%)	0 (0%)
	**Unknown**	0 (0%)	5 (6%)	0 (0%)	0 (0%)
Location of infection	**Pulmonary**	11 (37%)	‐	‐	51 (80%)
	**Extrapulmonary**	16 (53%)	‐	‐	9 (14%)
	**Both**	3 (10%)	‐	‐	1 (1%)
	**Unknown**	0 (0%)	‐	‐	3 (5%)
Culture confirmed	**Yes**	20 (67%)	‐	‐	64 (100%)
	**No**	10 (33%)	‐	‐	0 (0%)
Resistance	**None**	26 (87%)	‐	‐	‐
	**Isoniazid**	1 (3%)	‐	‐	‐
	**Pyrazinamide**	2 (7%)	‐	‐	‐
	**Rifampicin**	0 (0%)	‐	‐	‐
	**MDR**	1 (3%)	‐	‐	‐
NTM species	***Mycobacterium avium* complex**	‐	‐	22 (88%)	‐
	***Mycobacterium abscessus***	‐	‐	2 (8%)	‐
	***Mycobacterium malmoense***	‐	‐	1 (4%)	‐
Baseline ALT (IU/L)		18 (14–35)	15 (12–21)	15 (12–22)	20 (14–30)
Treatment	**Isoniazid, rifampicin**	‐	43 (48%)	‐	‐
	**Isoniazid**	‐	26 (30%)	‐	‐
	**Rifampicin**	‐	18 (20%)	‐	‐
	**Moxifloxacin**	‐	1 (1%)	‐	‐
	**Rifampicin, azithromycin**		‐	1 (4%)	‐
	**Rifampicin, clarithromycin**	‐	‐	3 (12%)	‐
	**Rifampicin, clarithromycin, amikacin**	‐	‐	1 (4%)	‐
	**Rifampicin, ethambutol**	‐	‐	1 (4%)	‐
	**Rifampicin, ethambutol, amikacin**	‐	‐	1 (4%)	‐
	**Rifampicin, ethambutol, clarithromycin**	‐	‐	15 (60%)	‐
	**Rifampicin, ethambutol, moxifloxacin**	‐	‐	1 (4%)	‐
	**Rifabutin, clarithromycin, moxifloxacin**	‐	‐	1 (4%)	‐
	**Clarithromycin, clofazimine, azithromycin**	‐	‐	1 (4%)	‐
	**Isoniazid, rifampicin, pyrazinamide, ethambutol plus antiretroviral therapy**	‐	‐	‐	64 (100%)
Initiation phase	**Isoniazid, rifampicin, pyrazinamide, ethambutol**	22 (73%)	‐	‐	‐
	**Isoniazid, rifampicin, pyrazinamide, moxifloxacin**	1 (3%)	‐	‐	‐
	**Isoniazid, rifampicin, pyrazinamide, ethambutol, moxifloxacin**	1 (3%)	‐	‐	‐
	**Isoniazid, rifabutin, pyrazinamide, ethambutol**	1 (3%)	‐	‐	‐
	**Isoniazid, rifampicin, ethambutol, moxifloxacin**	3 (10%)	‐	‐	‐
	**Rifampicin, ethambutol, moxifloxacin**	1 (3%)	‐	‐	‐
	**Bedaquiline, clofazimine, cycloserine**	1 (3%)	‐	‐	‐
Continuation phase	**Isoniazid, rifampicin**	20 (67%)	‐	‐	‐
	**Isoniazid, rifampicin, moxifloxacin**	2 (7%)	‐	‐	‐
	**Isoniazid, rifampicin, ethambutol**	2 (7%)	‐	‐	‐
	**Isoniazid, Rifabutin, moxifloxacin**	1 (3%)	‐	‐	‐
	**Isoniazid, ethambutol, moxifloxacin**	1 (3%)	‐	‐	‐
	**Isoniazid, rifampicin, pyrazinamide, ethambutol**	1 (3%)	‐	‐	‐
	**Rifampicin, ethambutol, moxifloxacin**	2 (7%)	‐	‐	‐
	**Bedaquiline, clofazimine, cycloserine**	1 (3%)	‐	‐	‐

ALT, alanine transaminase; HIV, human immunodeficiency virus; MiR, microRNA; NTM, nontuberculous mycobacterial; TB, tuberculosis

**TABLE 2:** Circulating ALT, miR‐122 and K18 in individuals with normal ALT (<50 IU/L) in healthy volunteers and first samples taken upon starting ALISTER study or SAEFRIF trial

		Healthy volunteers (*n =* 28)	Active TB (*n =* 26)	Latent TB (*n =* 87)	NTM infection (*n =* 25)	HIV‐TB coinfection (*n =* 59)
ALT (IU/L)	Median	18	22	16	16	18
	[IQR]	[12–22]	[14–27]	[12–23]	[13–25]	[14–27]
	Coefficient of variation (%)	44.0	40.7	48.4	54.3	50.3
MiR‐122 (fM)	Median	2.88	4.14	2.92	1.57	‐
	[IQR]	[1.75–3.96]	[1.34–7.56]	[1.46–4.54]	[0.91–3.72]	
	Coefficient of variation (%)	65.8	183.4	120.6	168.5	‐
K18 (U/L)	Median	210	145	152	163	172
	[IQR]	[134–259]	[92–220]	[105–215]	[138–263]	[132–276]
	Coefficient of variation (%)	45.9	62.2	207.8	69.2	79.4

ALT, alanine transaminase; HIV, human immunodeficiency virus; IQR, interquartile range; MiR, microRNA; NTM, nontuberculous mycobacteria; TB, tuberculosis.

**FIGURE 4:**
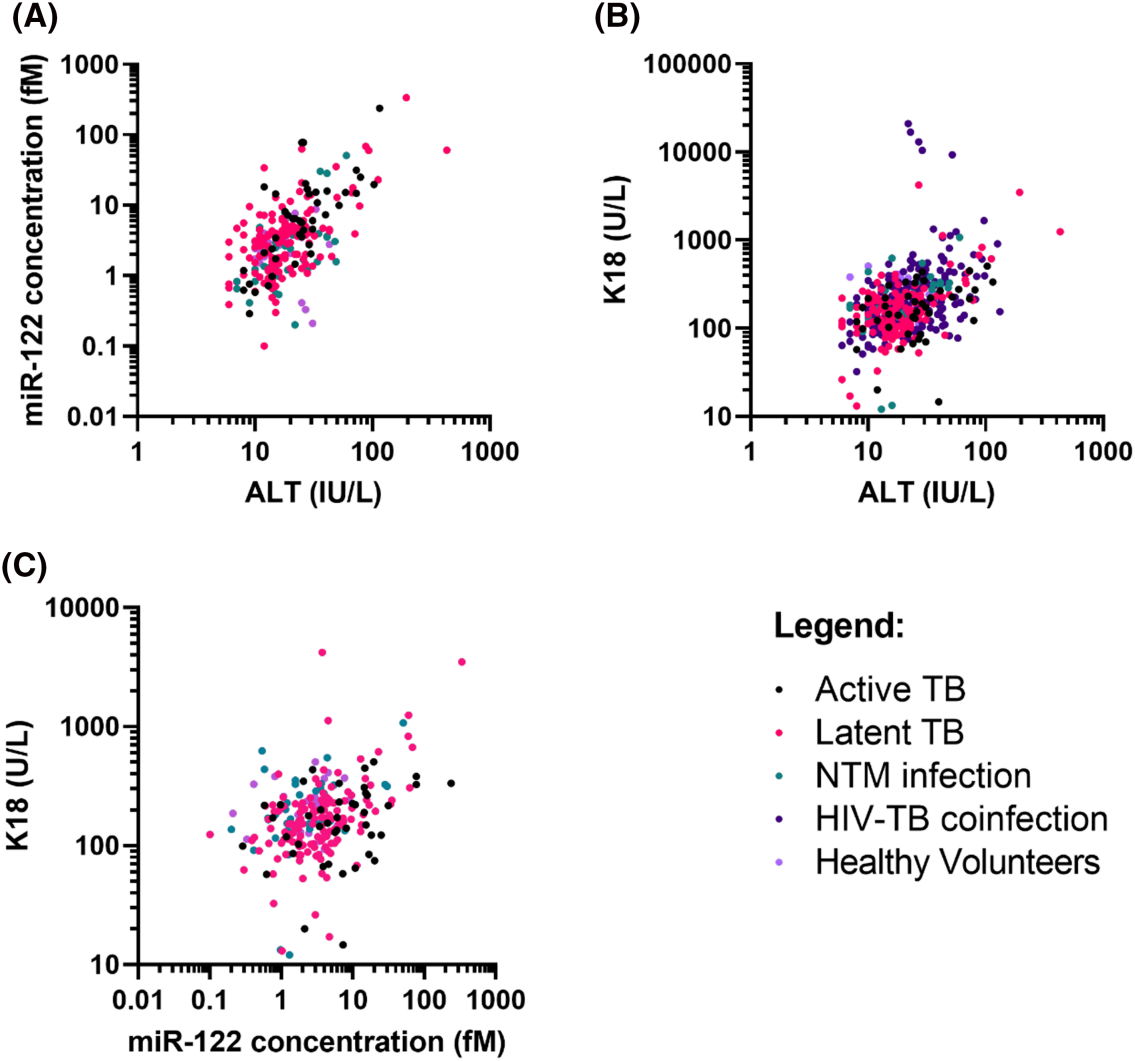
Correlation of A, microRNA (miR)‐122 (fM) *vs*. alanine transaminase (ALT; IU/L), B, K18 (U/L) *vs*. ALT (IU/L) and C, miR‐122 (fM) *vs*. K18 (U/L). Patient samples (healthy volunteers, *n =* 28; active tuberculosis [TB], *n =* 44; latent TB, *n =* 142; nontuberculous mycobacteria [NTM] infection, *n =* 39; human immunodeficiency virus–tuberculosis [HIV‐TB] coinfection, *n =* 241). Statistical analysis of the significance of the correlation calculated using Spearman's rank correlation coefficient (miR‐122 *vs*. ALT: *n =* 251, Spearman rank *R =* .52, 95% CI 0.42–0.61, *P <* .0001; K18 *vs*. ALT: *n =* 491, Spearman rank *R =* .42, 95% CI 0.34–0.49, *P <* .0001; miR‐122 *vs*. K18: *n =* 252, Spearman rank *R =* .32, 95% CI 0.20–0.43, *P <* .0001)

When all samples from all time points were included, there was a significant correlation between ALT and miR‐122 (*n =* 251, Spearman rank r = 0.52, 95%CI = 0.42–0.61, *P <* .0001; Figure 4A). There was a significant, but less tight, correlation between ALT and K18 (*n =* 491, Spearman rank r = 0.42, 95% CI = 0.34–0.49, *P <* .0001; Figure 4B). miR‐122 correlated with K18 (*n =* 252, Spearman rank r = 0.32, 95% CI = 0.20–0.43, *P <* .0001; Figure 4C).

**FIGURE 5:**
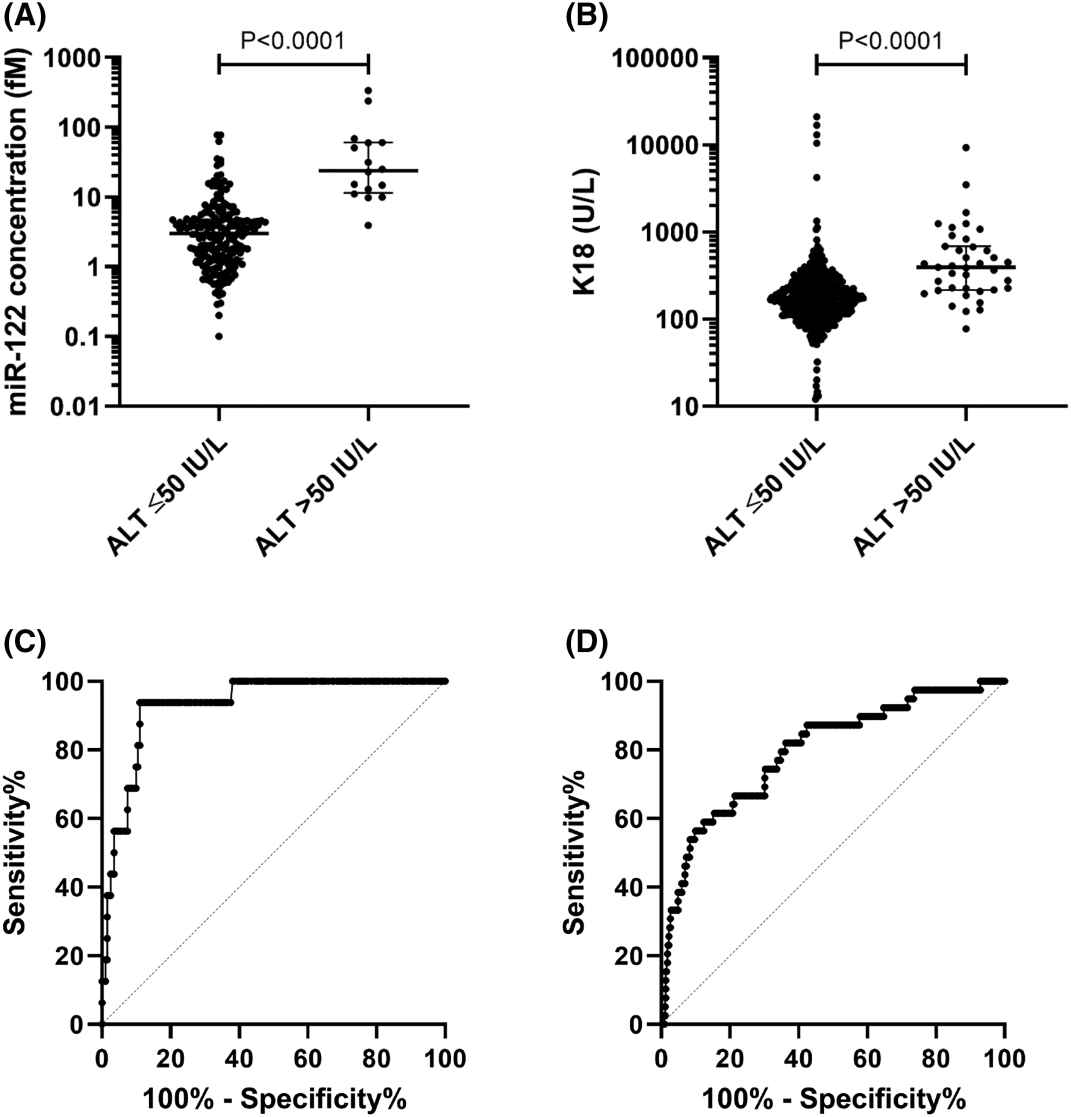
Comparison of samples grouped by normal alanine transaminase (ALT; ≤50 IU/L) and elevated ALT (>50 IU/L). A, MicroRNA (miR)‐122 concentration (fM) and B, K18 (U/L). Patient samples (healthy volunteers, *n =* 28; active tuberculosis [TB,] *n =* 44; latent TB, *n =* 142; nontuberculous mycobacteria infection, *n =* 39; HIV‐TB coinfection, *n =* 241). Statistical analysis of the significance of the difference between the groups calculated with the Mann–Whitney *t*‐test (miR‐122 *P <* .0001; K18 *P <* .0001). Receiver operator characteristic (ROC) analysis of samples grouped by normal ALT (≤50 IU/L) and elevated ALT (>50 IU/L), C, miR‐122 and D, K18. MiR‐122 (ROC‐area under the curve [AUC] = 0.93, 95% CI = 0.88–0.98, *P <* .001). K18 (ROC‐AUC = 0.80, 95% CI = 0.72–0.87, *P <* .0001)

MiR‐122 was increased 8.0‐fold in samples with elevated ALT (>50 IU/L) compared to samples with normal ALT (predefined as ≤50 IU/L; median [IQR]: elevated ALT 23.9 [11.5–60.4]; normal ALT 3.00 [1.31–4.82] fM; *P <* .0001; Figure 5A). K18 was increased 2.3‐fold in those samples with elevated ALT (>50 IU/L) compared to samples with normal ALT (≤50 IU/L; median [IQR]: elevated ALT 395 [217–683]; normal ALT 170 [120–250] U/L; *P <* .0001; Figure 5B). ROC analysis was performed on these grouped samples (Figure 5C,D). MiR‐122 identified elevated ALT (>50 IU/L) with high accuracy (ROC‐AUC = 0.93, 95% CI = 0.88–0.98, *P <* .001). K18 identified elevated ALT (>50 IU/L) with lower accuracy (ROC‐AUC = 0.80, 95% CI = 0.72–0.87, *P <* .0001). The sensitivity and specificity of miR‐122 and K18 was unchanged when only samples from patients on treatment were included in the analysis (miR‐122: ROC‐AUC = 0.93, 95%CI = 0.87 to 0.99, *P <* .0001. K18: ROC‐AUC = 0.78, 95%CI = 0.68 to 0.87, *P <* .0001; Figure S1).

The interindividual variability in miR‐122, K18 and ALT was compared in the different patient groups (Table 2). Interindividual variability was higher for both miR‐122 and K18 than ALT. Sequential samples from the SAEFRIF trial (patients with normal ALT (<50 IU/L) and 3 or more samples collected) were analysed to determine the intraindividual variability over time (CV median [IQR]: ALT 23.9 [16.6–36.1] %; K18 35.4 [24.9–41.9] %; *P =* .02). Intraindividual variability was higher for K18 than ALT.

**FIGURE 6:**
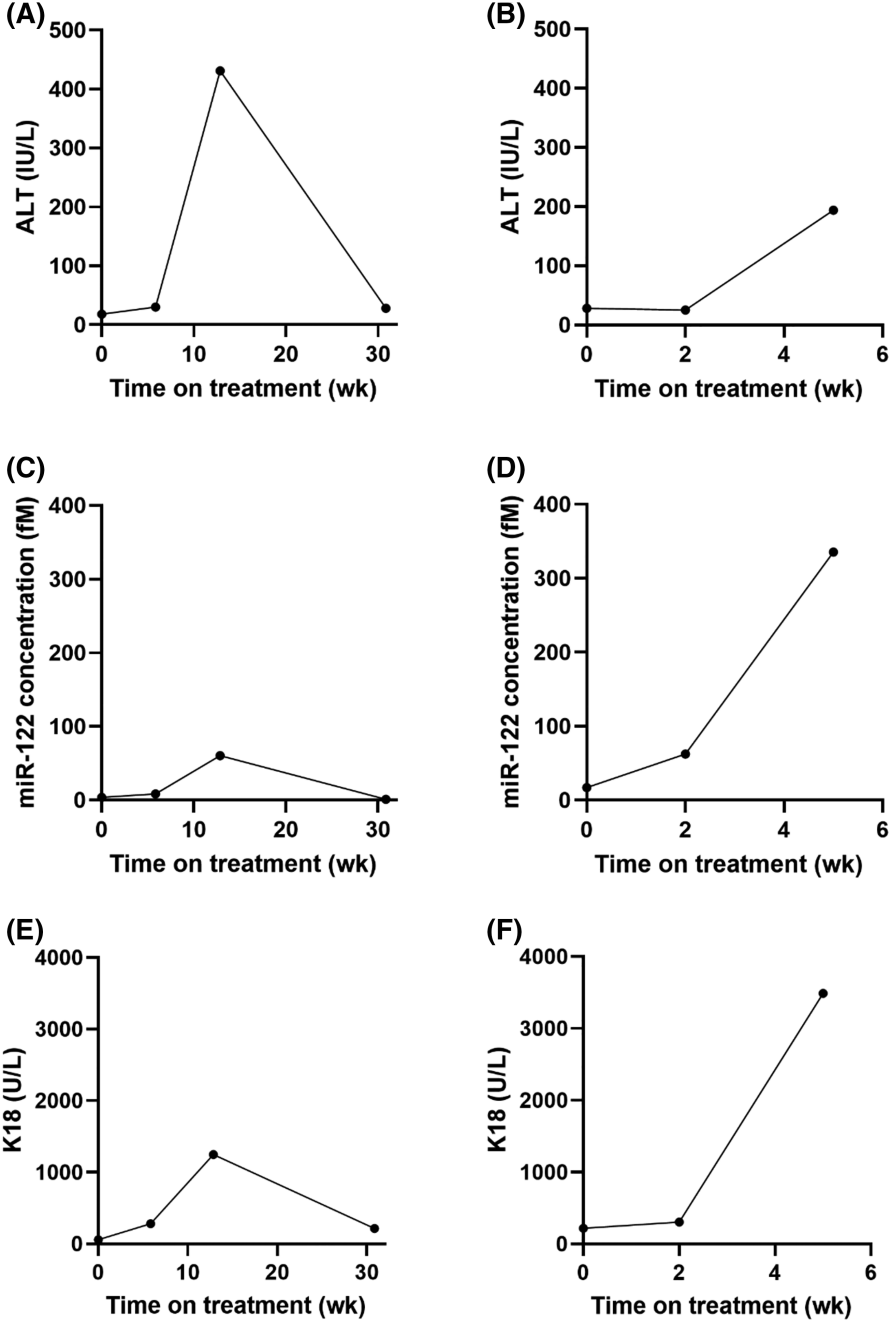
Circulating concentration of (A, B) alanine transaminase (ALT; IU/L), (C, D) microRNA (miR)‐122 (fM) and (E, F) K18 (U/L) over the course of treatment (weeks) for 2 cases who developed DILI as predefined as >3× upper limit of normal ALT in the presence of symptoms or >5× upper limit of normal in absence of symptoms.^8^ Case 1 (A, C, E); Case 2 (B, D, F)

In this study there were 2 cases of DILI (as predefined in study protocols), both cases were for patients receiving isoniazid alone for the treatment of latent TB within the ALISTER study. The first case was of a 51‐year‐old white British male patient, who experienced peak ALT activity of 431 IU/L, (Figures 6A,E). Before starting treatment, he had normal ALT (18 IU/L), miR‐122 was 4 fM and his K18 was 58 U/L. Three months into treatment, his ALT activity increased to 431 IU/L, miR‐122 rose to 60 fM and K18 rose to 1248 U/L. Drug treatment was halted and ALT returned to within normal limits 18 weeks later. Formal causality between isoniazid and liver injury was determined using the RUCAM scale and was determined as probable (RUCAM scale = 6).

The second case was of a 71‐year‐old white British female patient, **(**Figures 6B,D,F **).** At baseline her ALT activity was 28 IU/L, miR‐122 was 17 fM and K18 was 219 U/L. Two weeks into treatment, her ALT activity had not increased (25 IU/L) but K18 and miR‐122 had risen (307 U/L and 63 fM, respectively). DILI was present 5 weeks into treatment with the presence of a drug rash and elevated ALT at 194 IU/L. At this time, K18 activity had risen to 3490 U/L and miR‐122 concentration had increased further to 336 fM. Drug treatment was halted, and the patient was discharged and lost to further follow up. RUCAM causality assessment indicated that isoniazid was the probable cause of liver injury (RUCAM scale = 7).

## 4 DISCUSSION

The utility of miR‐122 and K18 as exploratory DILI biomarkers has been demonstrated by several multicentre studies and this has resulted in Food and Drug Administration support for continued development to full qualification.^21^ However, the properties of these biomarkers have not been robustly studied in patients with TB in Africa.

Given the potential involvement of the liver in TB infection, it was important to determine if circulating concentrations of miR‐122 and K18 differed from healthy individuals in the presence of infection, including active, latent and NTM infection and HIV‐TB coinfection. For example, if either biomarker was elevated by mycobacterial infection *per se* then it would be de‐prioritised as a biomarker in this important context of use. In this study, we have demonstrated that circulating miR‐122 and K18 in healthy volunteers and patients with active TB, latent TB and NTM infection are not substantially different. This suggests that the presence of mycobacterial infection does not affect circulating miR‐122 and K18. Furthermore, circulating K18 in HIV‐TB coinfected African patients was similar to the other groups, which were predominantly Caucasian. This suggests that the healthy reference interval for K18 in an African and Caucasian population is likely to be similar. In addition, we have demonstrated that, in the absence of DILI, neither miR‐122 nor K18 change substantially upon commencing treatment. Both miR‐122 and K18 correlate with ALT, indicating these biomarkers may have diagnostic utility. In this pilot study, miR‐122 distinguished those patients with an elevated ALT with greater sensitivity and specificity than K18 but this should be interpreted with caution as there were only 2 cases of DILI as predefined in our study protocol. In these DILI cases, the elevations in ALT temporarily correlated with a rise in both miR‐122 and K18. In addition, in 1 of these patients, miR‐122 and K18 rose before ALT, indicating a potential for these novel biomarkers to predict the development of DILI earlier than ALT. The results of this study provide initial evidence for the potential use of both miR‐122 and K18 as biomarkers of TB medicine associated DILI.

Further work should focus on determining the diagnostic value of the biomarkers, whether they correlate with rises in ALT and so can diagnose DILI within this population. A clear definition of the dynamic range, sensitivity and specificity of miR‐122 and K18 within this population is needed before they can be used as a biomarker of DILI. Furthermore, given that evidence suggests miR‐122 and K18 both rise earlier than ALT in paracetamol DILI, it is important to determine if they have the same predictive value in patients with mycobacterial infections. This predictive ability of these novel biomarkers may enable early identification of patients at risk of DILI, leading to prevention of liver injury through halting or altering treatment regimens before significant liver injury develops. Specifically, the biomarkers could be a useful early indicator of the development of DILI in patients being reintroduced to essential anti‐TB medications, a group at elevated risk of DILI recurrence.

Our study had a limited number of cases of anti‐TB DILI. Historical data suggested approximately 2–5% of patients receiving anti‐TB treatment in the UK will develop DILI. However, within the ALISTER study only 2 patients developed DILI, 1.4% of the patients recruited. Larger multicentre studies are required to recruit enough patients to determine the diagnostic power of miR‐122 and K18 in anti‐TB DILI. The majority of values for circulating miR‐122 concentrations in the patient groups fell within the published upper limit of the healthy reference interval of 45 fM generated from the SAFE‐T dataset.^34^ However, there were 2 patients with miR‐122 increased above this healthy reference interval (miR‐122 = 77 and 77 fM) when ALT was still normal (ALT = 25 and 26 IU/L). This may reflect a limitation of miR‐122, namely that it has been reported to have relatively high variability.^34^ In our study, the variability of the novel biomarkers was higher than ALT, with miR‐122 having higher intersubject variability than K18. Although the previously published healthy reference interval provides a valuable comparison, the circulating concentration of miR‐122 in healthy volunteers in this study fell between 0.21 and 8.75 fM, considerably lower than the published ULN of 45 fM, which was generated from the SAFE‐T dataset.^34^ This healthy reference interval was developed using a larger sample size than that included in this study. However, it was determined using different quantification and normalisation methods, therefore a direct comparison is challenging. There were 2 patients who had substantially elevated K18 in the absence of elevated ALT. Firstly, in ALISTER (K18 = 4207 U/L, ALT = 43 IU/L). Secondly, in the SAEFRIF trial, where a patient had K18 ranged from 10 000–20 000 U/L, but not substantially elevated ALT (22–52 IU/L). The reason for these 2 outliners is unknown and requires further study with larger patient numbers. Another limitation of our study is that miR‐122 was only measured in the ALISTER cohort, whereas K18 was measured in both ALISTER and SAEFRIF cohorts. This limitation was due miR‐122 only being measured in Edinburgh. The global COVID pandemic and the HIV‐positive status of the SAEFRIF samples meant these could not be transferred out of Uganda. Given the possible superior sensitivity/specificity of miR‐122 over K18 which is suggested by this pilot study it will be important for future studies to measure miR‐122 in the African setting. Finally, this study did not include patients recruited in Asia, a region with high TB prevalence. This group should be included in future studies.

In summary, the presence of mycobacterial infection does not alter miR‐122 or K18 concentrations in the absence of DILI. African HIV‐TB coinfected patients had similar K18 concentrations to healthy volunteers and Caucasian TB patients. Patients who experienced elevations in ALT also demonstrated rises in both miR‐122 and K18 indicating the diagnostic potential of these biomarkers. Future trials of miR‐122 and K18 as biomarkers of anti‐TB DILI could be performed using the data presented in this paper to inform the study design.

## CONFLICT OF INTEREST

The authors declare no conflicts of interest.

## Supporting information


**FIGURE S1** Comparison of samples grouped by normal ALT (≤50 IU/L) and elevated ALT (>50 IU/L) in those patients on treatment for mycobacterial infection. (A) miR‐122 concentration (fM) and (B) K18 (U/L). Statistical analysis of the significance of the difference between the groups calculated with the Mann–Whitney test (miR‐122 *P <* .0001; K18 *P <* .0001). ROC analysis of samples grouped by normal ALT (≤50 IU/L) and elevated ALT (>50 IU/L), (C) miR‐122 and (D) K18. ROC, receiver operator characteristic; AUC, area under the curve.

## Data Availability

Data available on request from the authors.
