# Supplementary figures and images for: MicroRNA‐122 and cytokeratin‐18 have potential as a biomarkers of drug‐induced liver injury in European and African patients on treatment for mycobacterial infection
Source: Br J Clin Pharmacol. 2021 Jan 26;87(8):3206–17. doi: 10.1111/bcp.14736 (PMC8629110; doi:10.1111/bcp.14736)

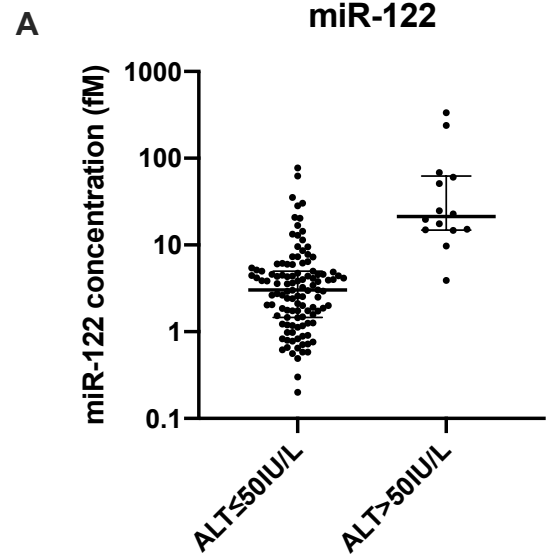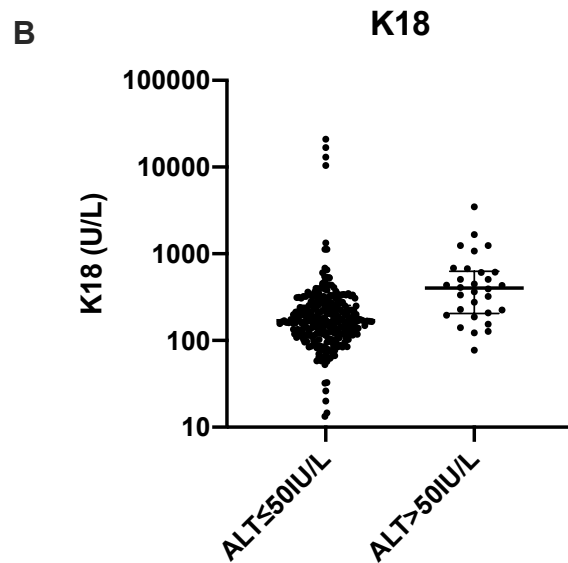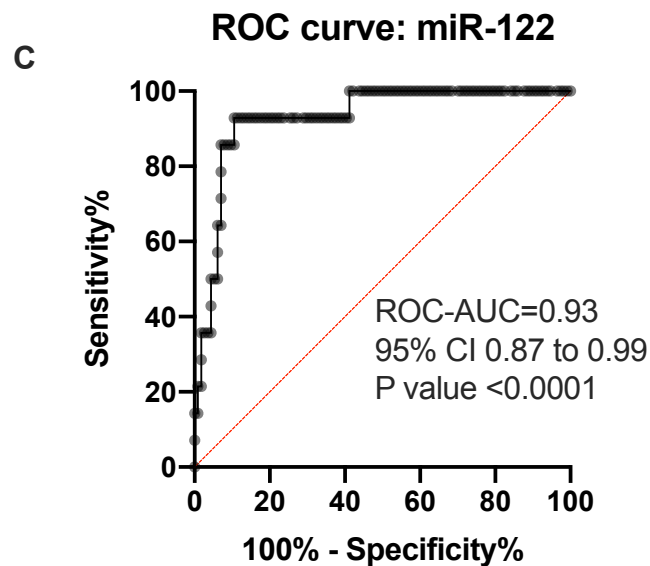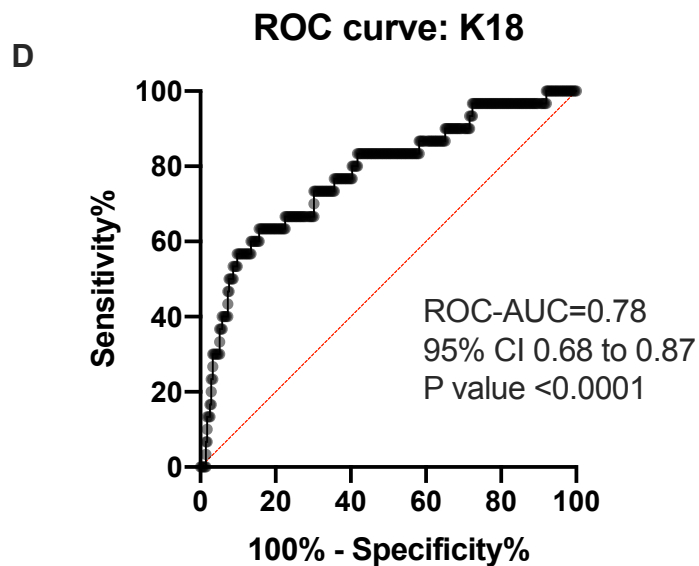

Supplement: Supplementary file 1 — FIGURE S1 Comparison of samples grouped by normal ALT (≤50 IU/L) and elevated ALT (>50 IU/L) in those patients on treatment for mycobacterial infection. (A) miR‐122 concentration (fM) and (B) K18 (U/L). Statistical analysis of the significance of the difference between the groups calculated with the Mann–Whitney test (miR‐122 P < .0001; K18 P < .0001). ROC analysis of samples grouped by normal ALT (≤50 IU/L) and elevated ALT (>50 IU/L), (C) miR‐122 and (D) K18. ROC, receiver operator characteristic; AUC, area under the curve. [file BCP-87-3206-s001.pdf]
